# Saccharomyces cervisiae ameliorative impact combined with sulfaclozine on broiler chicken oxidative status

**DOI:** 10.1186/s12917-025-04955-x

**Published:** 2025-08-06

**Authors:** Nahla M. Ali, Mohamed K. Hussein, Nady Khairy Elbarbary, Zeinab-El Amgad, Enas A. Noseer

**Affiliations:** 1 Department of Biochemistry, Faculty of Veterinary Medicine, Aswan University, Aswan, 81528 Egypt; 2 Department of Food Hygiene and Control, Faculty of Veterinary Medicine, Aswan University, Aswan, 81528 Egypt; 3 General Authority for Veterinary Services, Qena Veterinary Directorate, Qena, Egypt

**Keywords:** Broiler chicken, Sufaclozine, *Saccharomyces cervisiae*, Biochemical parameter, Histopathology

## Abstract

Poultry production has expanded, and imports from other countries have surged due to the sharp growth in demand for chicken meat. The purpose of this study was to determine the effect of different doses of sulfaclozine 30%, either alone or in conjunction with Saccharomyces cerevisiae (yeast), on body weight gain, biochemical markers, oxidative status, and histopathological variation in broilers’ organs. One hundred fourteen -day-old chicks were divided into five groups for this experiment (20 each). For a total of 20 days, the first group was used as a control; the second group received 30 mg/kg of sulfaclozine; the third group received 70 mg/kg; the fourth group received 1.5 g/L of *Saccharomyces cerevisiae* followed by 30 mg/kg of sulfaclozine; and the final group received 70 mg/kg of sulfaclozine after taken 1.5 g/L of *Saccharomyces cerevisiae*. Hepatic and renal functions, along with malonaldehyde levels, were much higher in the chickens that received 70 mg/kg of sulfaclozine followed by 30 mg/kg; however, total protein, albumin, and catalase levels were much lower, and signs of liver and kidney damage were seen. On the other hand, the groups that received sulfaclozine treatments subsequent *Saccharomyces cerevisiae* showed improved metabolic indices and a partial recovery from the histopathological change.

**Supplementary Information:**

The online version contains supplementary material available at 10.1186/s12917-025-04955-x.

## Introduction

One of the largest food industries worldwide is the chicken industry. Food production systems like the poultry industry have shown an exponential rise in global meat consumption and business profit [1]. Antibiotics are essential bio control tools strategically applied in contemporary animal and poultry systems as growth promoters, therapeutic interventions, and disease prevention and control measures [2]. However, the extensive use of antibiotics has caused an antibiotic residue problem in poultry meat and increased antibiotic-resistant bacteria’s proportion and persistence [3]. Sulfonamides are relatively old synthetic antibacterial compounds still effective in treating cecal coccidiosis in poultry [4]. Natural compounds, such as probiotics, have been used to counteract the negative effects of antibiotics and reduce their adverse effects. Administering a probiotic concurrently with an antibiotic can reduce the risk of symbiosis and other antibiotic-related problems associated with gut bacteria, such as inflammation, yeast overgrowth, diarrhea, and superinfections [5]. Probiotics contain an antioxidant system that comprises catalase and superoxide dismutase (SOD) [6].

Additionally, probiotics generate several metabolites associated with the antioxidant system, such as B vitamins, butyrate, and glutathione. Folate is a key in DNA replication, repair, and methylation efficiency [7]. Probiotics help control antioxidant enzymes in the body, like glutathione peroxidase, catalase, superoxide dismutase, and glutathione-S transferase, to boost antioxidant benefits [8]. The current study aims to document the protective effect of Saccharomyces cerevisiae (*S. cerevisiae*) against sulfaclozine’s toxic effects on broiler chickens’ liver and kidneys.

## Materials and methods

### A-Chemicals

Sulfaclozine (Atcocure) sulfonamide derivative was purchased from Atco Pharma. Cairo, Egypt. *Saccharomyces cerevisiae* (yeast) was purchased from Beni Suef, an Angel Yeast Company in Egypt.

### Reagents

Kits of hepatic function, renal function, total protein, and albumin were obtained from Spectrum Egyptian Company for Biotechnology. Malonaldehyde (MDA) and catalase enzyme kits were obtained from Bio-Diagnostic Company Dokii Giza, Cairo, Egypt. The RNA extraction kit, cDNA reverse transcription kit, and PCR master-mix were purchased from Applied Biotechnology, Ismailia, Egypt. Primers for targeted nuclear factor erythroid 2-related factor 2 (NRF2) and transforming growth factor beta 1 (TGFβ1) genes were synthesized by Metabion International AG Germany. Positive control samples of NRF2 and TGFβ1 were obtained from the Reference Lab for Safety Analysis of Foods of Animal Origin. Animal Health Research Institute, Agriculture Research Center, Giza, Egypt.

### Animals

One hundred male Ross broiler chicks, 14 days old, were sourced from the Qena company farm in Egypt. Broiler chicks were reared in separate cages, which were also cleaned daily to avoid infection, and contaminated water and food were available ad libitum with a suitable temperature and lighting cycle of 12 h/dark. At 21 days of age, we fed the treatment diet to the chickens. All experimental procedures were performed in accordance with the international guidelines for the care and use of laboratory animals.

### Experimental design

One hundred male Ross broilers were divided into five groups, with 20 birds in each group:

#### G1

(Negative control): Fed a standard diet.

#### G2

Received oral administration of Sulfaclozine at 30 mg/kg body weight [9, 10].

#### G3

Received oral administration of Sulfaclozine at 70 mg/kg body weight [11].

#### G4

Received 1.5 g/liter of *S. cerevisiae* added to their drinking water [12]followed by Sulfaclozine at 30 mg/kg body weight.

#### G5

Received Sulfaclozine at 70 mg/kg body weight after 1.5 g/liter of *S. cerevisiae* added to their drinking water.

### Serum and plasma collection

Blood samples were collected from the brachial vein using a syringe. Samples were taken at 26, 31, 36, and 41 days old in clean test tubes. Some samples were used to obtain serum, while others were used for plasma. The samples were then centrifuged at 3000 rpm for 20 min. Serum and plasma were separated and stored at -20 °C for use in the determination of biochemical parameters.

### Tissue collection

Chicks from each group were sacrificed at 26, 31, 36, and 41 d of age. The liver and kidney of each chick were dissected and preserved in 10% buffered formalin for fixation and histopathological examination. Specimens from the liver were stored at -80 °C to detect gene expression related to oxidative stress responses using the conventional polymerase chain reaction technique.

### Body weight

Chickens were weighed at the beginning of the study (0 times) and again on days 26, 31, 36, and 41.

### Determination of the serum biochemical parameters

#### Liver and kidney function

The tests for serum alanine aminotransferase (ALT; Cat. No. 2650052), serum aspartate aminotransferase (AST; Cat. No. 260002), total protein (Cat. No. 310001), albumin (Cat. No. 2110001), renal function tests for uric acid (Cat. No. 320004), and creatinine (Cat. No. 2370002) were done following the instructions from the manufacturer, Spectrum.

### Determination of oxidative parameter

MDA (Cat. No. 2529) and catalase (Cat. No. CA2517) were determined according to the manufacturer’s instructions using Bio-Diagnostic kits (Giza, Egypt).

### Histopathological examination

For histopathological studies, each chick’s liver and kidney tissue samples were excised and processed for light microscopy. The processing involved fixing The tissue samples were preserved in a 10% neutral buffered formalin solution, embedded in paraffin, sliced into 5 μm thick sections, and colored with hematoxylin and eosin (H&E) [13].

### Detection of TGFβ1 and NRF2 by PCR

According to the manufacturer’s instructions, the ABT Total RNA Mini Extraction Kit (Cat. no. ABT002, Applied Biotechnology, Egypt) was used to extract total RNA from the samples. cDNA was made using a cDNA Reverse Transcription Kit (ABT 2X RT Mix Oligo dt 18, Cat. no. AMP11, Applied Biotechnology, Egypt) following the manufacturer’s instructions to check the gene expression of TGFβ1 [14] and NRF2 [15] with the primer sequences listed in Table 1.

PCR amplification was achieved in a 25 µl reaction volume using 12.5 µl of ABT 2X Red Mix, 1 µl of each primer, 1 µl of extracted DNA, and 25 µL of nuclease-free water. The PCR products were separated by electrophoresis on a 1.5% agarose gel (AppliChem GmbH, Germany) in 1x TBE buffer at ambient temperature. Gradients of 5 V/cm were applied, and 15 µL of the products were loaded into each gel slot. The resultant gel was stained with 0.5 µg/ml Ethidium bromide and then visualized using a UV trans-illuminator and digital camera photography [16].

**Table 1:** The specific sequences and amplified products of oligonucleotide primers used in the PCR technique

Primer	Oligonucleotide (5’-3’)	Amplicon (bp)
*NRF2*	F: CTGCCCAAAACTGCCGTA	285 bp
	R: TCAAATCTTGCTCCAGTTCCA	
*TGFβ1*	F: GACGATGAGTGGCTCTCCTTC	195 bp
	R: GTGCTTCTTGGCAATGCTCT	

### Statistical analysis

Results are presented as mean ± SD. Variation within a data set was analyzed by one-way analysis of variance (ANOVA) using Graph Pad Prism Software (GPPS). Statistical significance was set at *p* < 0.05%.

## Results

### Body weight

**Fig. 1:**
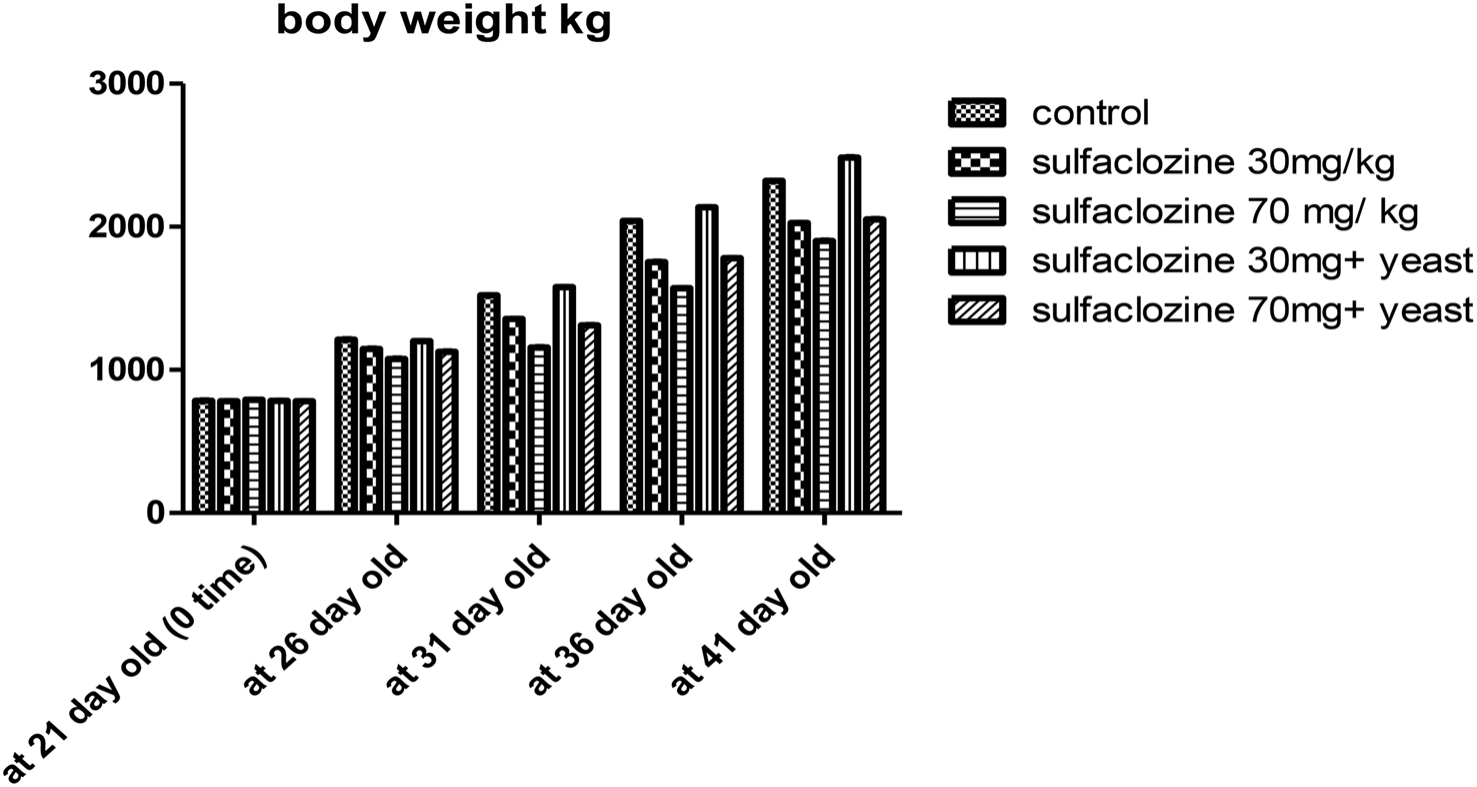
Body weight for G1, G2, G3, G4, and G5 throughout the experimental period

The effect of sulfacalozine at a dosage of 70 mg/kg (G3) showed a substantial decrease in body weight compared to the control group, G2, G4, and even G5 at 31, 36, and 41 days of age. In contrast, G4 significantly improved total body weight compared to G2, G3, G5, and the control group, as shown in Fig. 1.

**Fig. 2:**
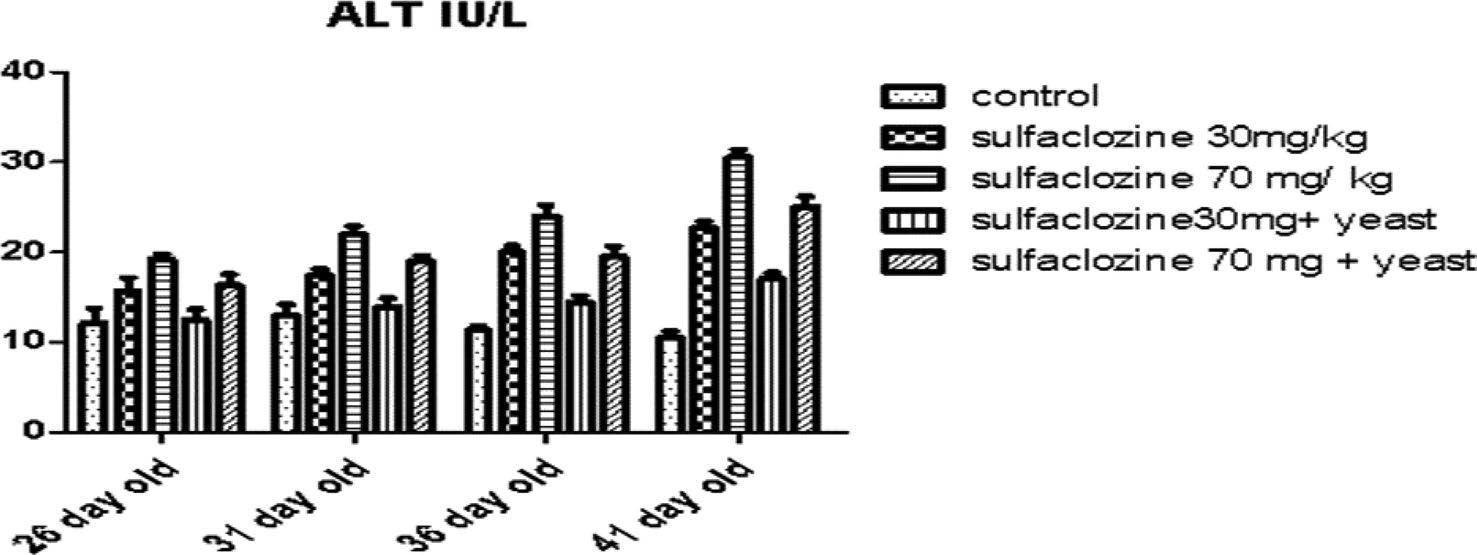
ALT for G1, G2, G3, G4, and G5 throughout the experimental period

Figure 2 shows a significant increase in ALT levels in all groups at 26, 31, 36, and 41 days of age compared to the control, except for G4, which significantly improved hepatic function.

**Fig. 3:**
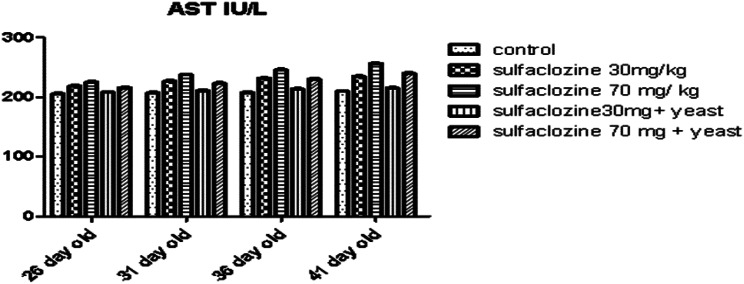
AST for G1, G2, G3, G4, and G5 throughout the experimental period

Figure 3 shows a non-significant increase in G2, G3, and G5 at 26, 31, and 36 days of age compared to the control group; however, at 41 days of age, there is a significant increase in G3 and G5 compared to all groups, while G4 shows a normal level similar to G1.

**Fig. 4:**
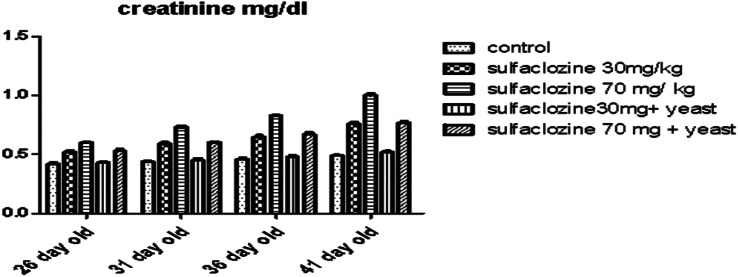
Creatinine for G1, G2, G3, G4, and G5 throughout the experimental period

Renal function impersonated in Fig. 4 shows a significant increase in creatinine in G3 from 31- to 41-day-olds compared to the control group and G4. In contrast, G2 and G5 reported a substantial increase only at 41 days and a non-significant increase from 26 to 36 days, which was not significantly different from G4, and G1.

**Fig. 5:**
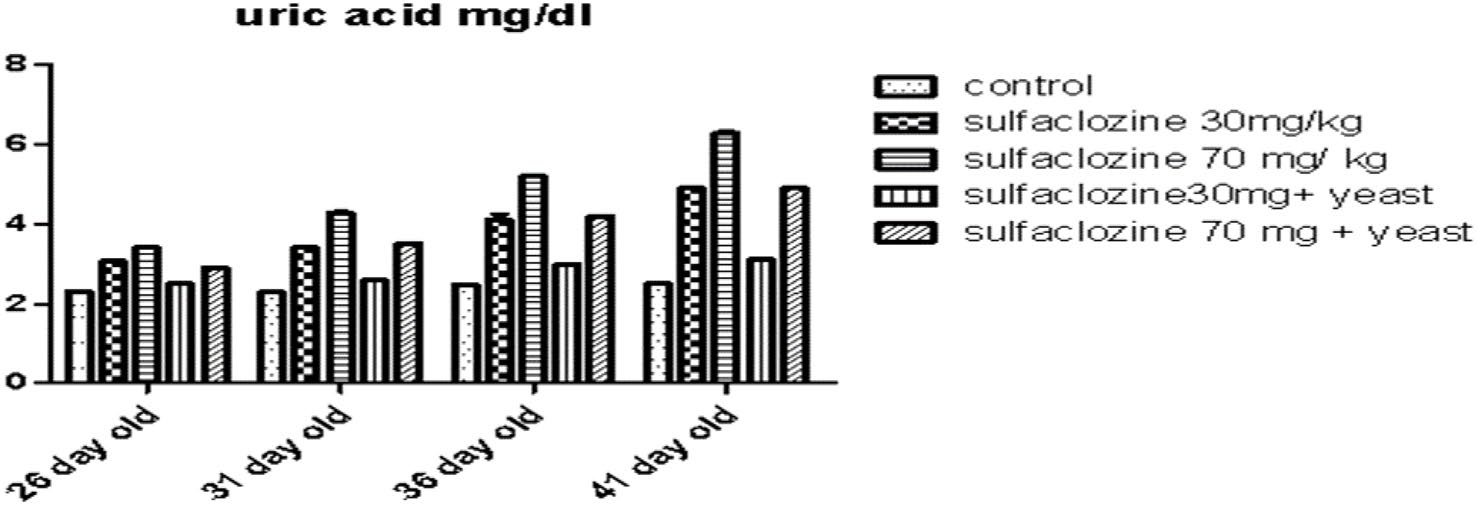
Uric acid for G1, G2, G3, G4 and G5 throughout the experimental period

Concerning uric acid levels (Fig. 5) at 26 days, chickens show a non-significant rise in G3, G2, and G5 compared to G1, and G4. However, G3 increased progressively, followed by G2, and G5, respectively, compared to control and G4 at 31, 36, and 41 days.

**Fig. 6:**
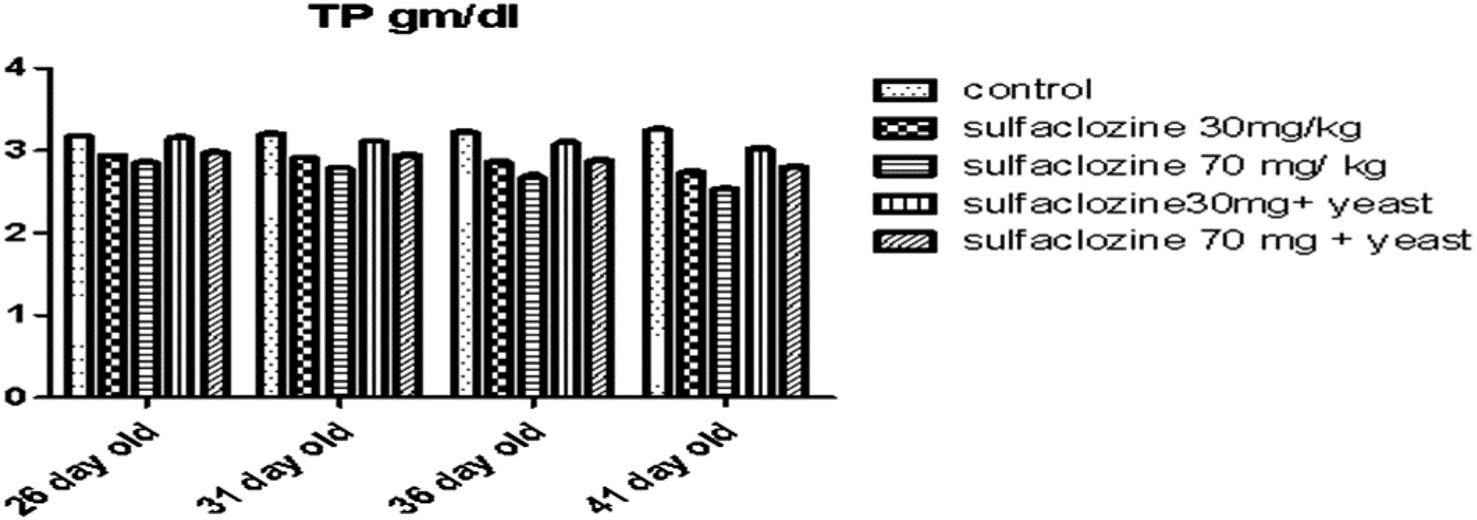
Total protein for G1, G2, G3, G4, and G5 throughout the experimental period

Besides, G4 recorded a significant increase in total protein (Fig. 6) from 36 to 41 days, compared to G2, G3, and G4. From 26 to 41 days old, G1 demonstrated a notable rise compared to G2, G3, and G5.

**Fig. 7:**
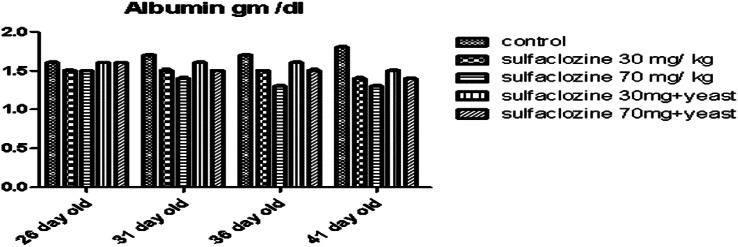
Albumin for G1, G2, G3, G4, and G5 throughout the experimental period

Albumin levels (Fig. 7) were not significantly reduced at 26 days of age in all exposed groups (G2, G3, and G5 when compared with the control), while G4 displayed significant improvement in emulation to G2, G3, and G5 at 31, 36, and 41 days of age; G1 highlighted the higher level of albumin (31–41 days old) compared to other treated groups.

**Fig. 8:**
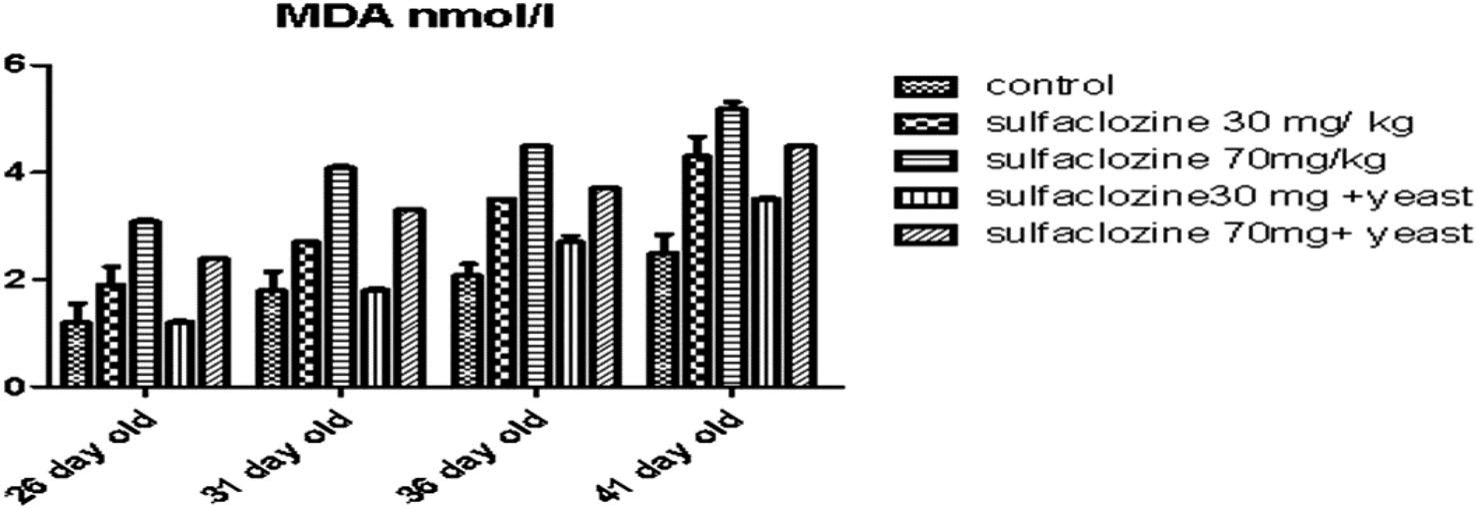
MDA for G1, G2, G3, G4, and G5 throughout the experimental period

Figure 8 in all receiving groups, aside from G4, showed a significant increase in MDA levels compared to the control group, especially G3 from 26- to 41-day-old chickens. On the other hand, G4 is similar to G1 in achieving low levels of MDA till the end of the experiment.

**Fig. 9:**
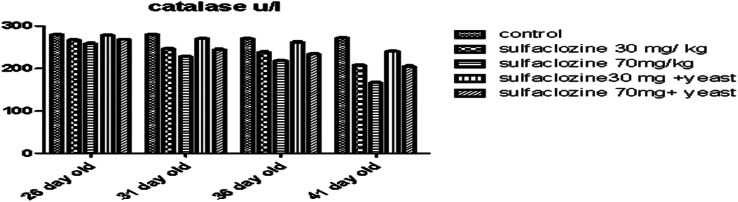
Catalase enzyme for G1, G2, G3, G4, and G5 throughout the experimental period

Compared to the control, the catalase activity of all exposed groups, except G4, was considerably reduced from 31 to 41 days of age, as shown in Fig. 9.

### Reverse transcriptase PCR

PCR electrophoresis of TGFβ1, and NRF2 genes in chicken liver M: 100 bp ladder. Cn: negative control, Cp: positive control e for the TGFβ1, and NRF2 t 285 bp. Lane: 1 and 2: control samples. Lane 3 and 4: treated group with Sulpha 30 mg. Lane 5 and 6: treated group with Sulpha 70 mg. Lane 7 and 8: treated group with Sulpha 30 mg and Saccharomyces. Lane 9 and 10: treated group with Sulpha 70 mg and Saccharomyces.

At 36 and 41 days, the mRNA expression of TGFβ1 in chicken liver is much lower in G4 and G5 and significantly higher in G2 and G3. As seen in Figures A (26 days), B (31 days), C (36 days), and D (41 days). But as Figures illustrate. NRF2 gene expression in chicken liver was higher in G4 and G5 and lower in G2 and G3 for E (26 days), F (31 days), G (36 days), and H (41 days). (supplementary files)

### Histopathological examination

#### Liver

**Fig. 10:**
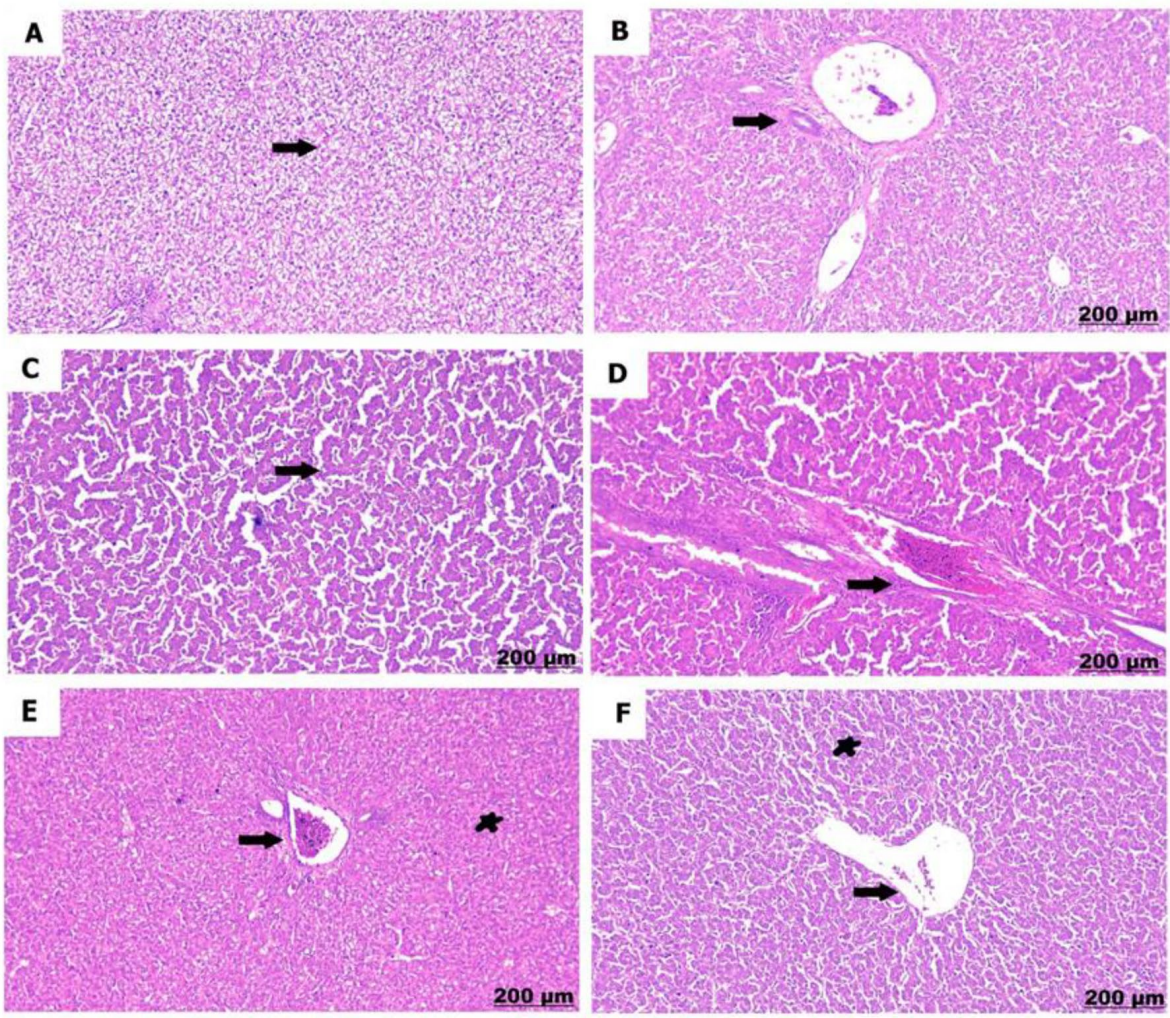
5 days post treatment. **A**) shows normal arranged hepatic cords. **B**) shows dilatation and proliferation of portal area. **C**) shows hepatic disorganization and necrosis. **D**) shows fibrosis and hypercerullarity of the bile duct. **E**) shows mild congestion of the portal area (arrow) with slight hepatocytes degeneration (star). **F**) shows congestion and dilatation of central vein (arrow), hepatic vacuolation (star)

**Fig. 11:**
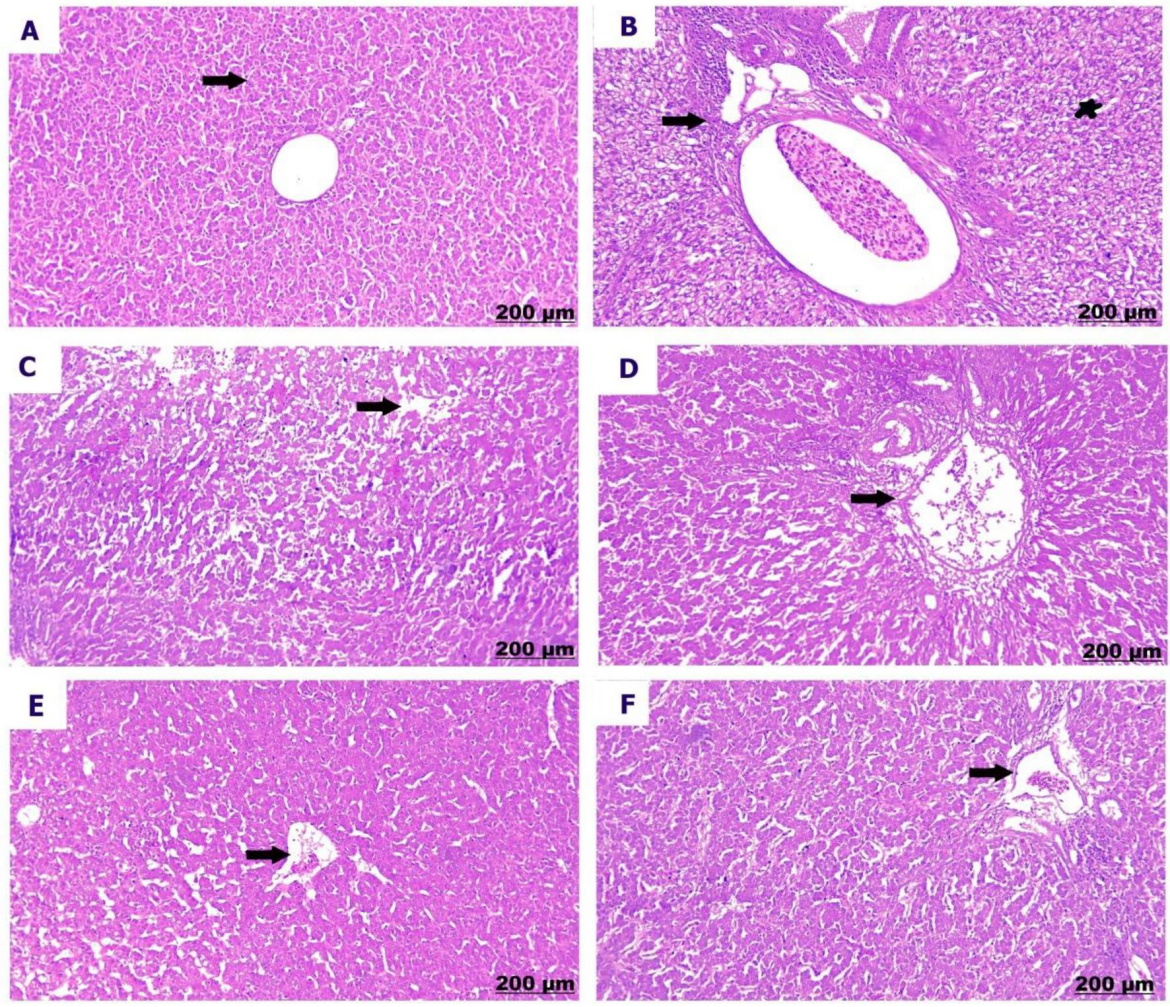
10 days post treatment. **A**) shows intact hepatic tissues. **B**) shows severe proliferation of portal area. **C**) shows severe lytic necrosis of the hepatocytes. **D**) shows proliferation and thickening of the bile duct. **E**) shows slight congestion of the blood vessels. **F)** shows thickening and congestion of central vein

**Fig. 12:**
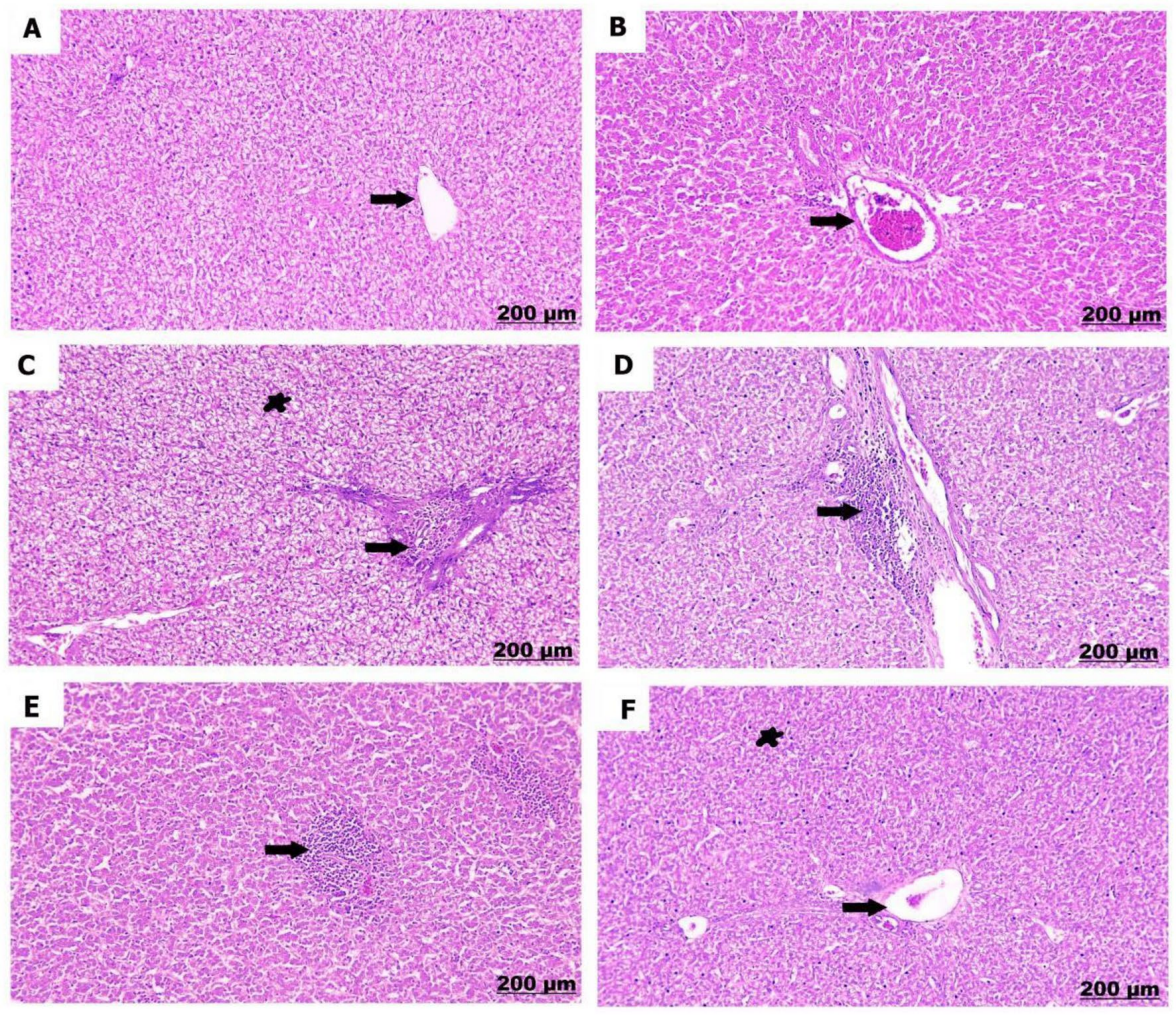
15 days post treatment. **A**) shows healthy blood vessels. **B**) shows congestion and periportal inflammation. **C**) shows vacuolation of the hepatocytes (star), besides thickening of the wall of the portal vessels (arrow). **D**) shows severe periportal infiltration. **E**) shows focal accumulation of the lymphocytes. **F**) shows mild congestion of blood vessels

**Fig. 13:**
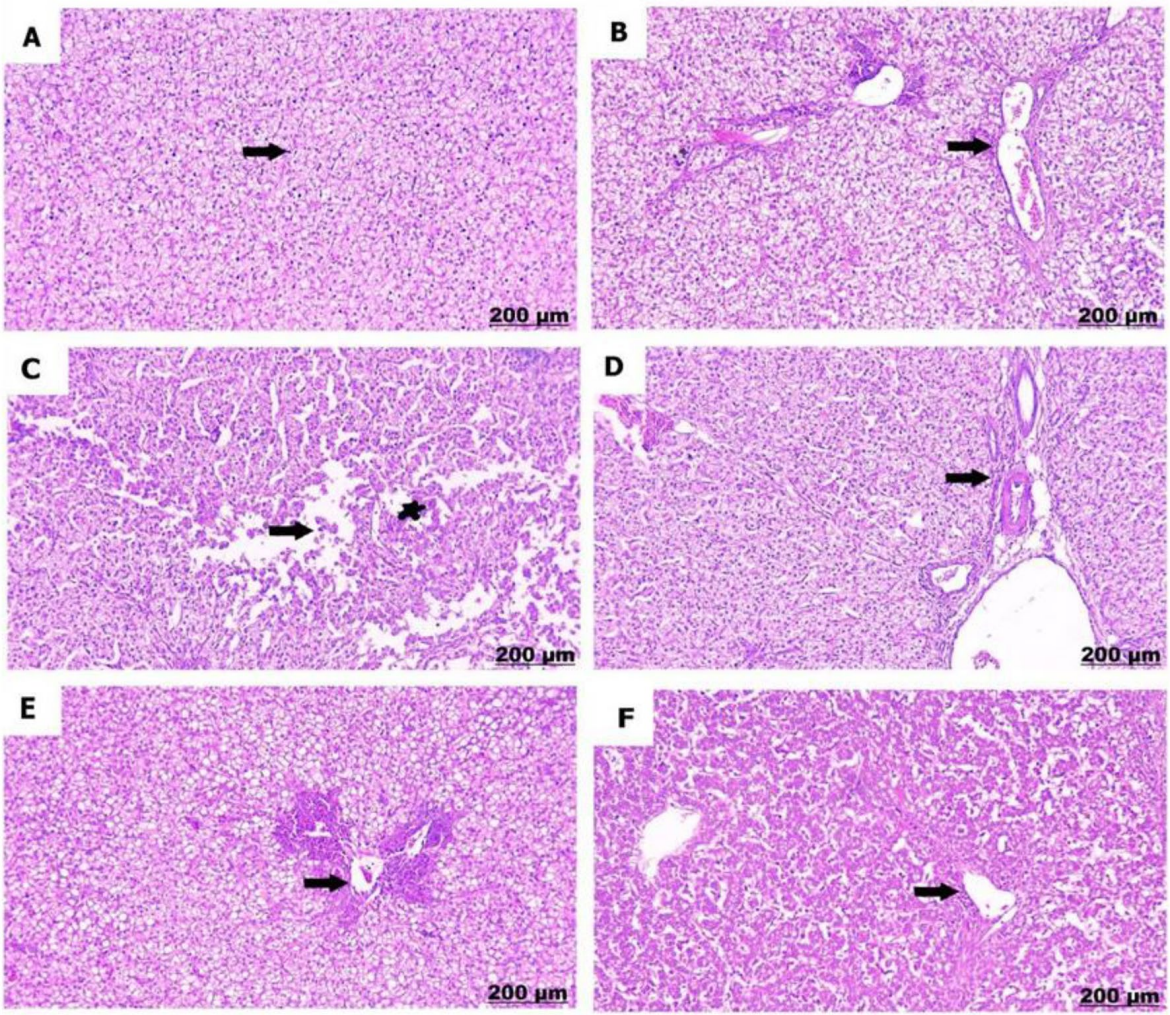
for 20 days post treatment. **A**) shows well organization of the hepatocytes. **B**) shows congestion and thickening of portal vessels. **C**) shows severe necrobiotic changes of the hepatocytes (arrow), and necrosis of the central vein (star). **D**) shows periportal fibrosis. **E)** shows portal congestion and inflammation. **F**) shows minimal congestion of blood vessels

The control chicks (A) showed normal liver structure with liver cells arranged properly and healthy blood vessels. Conversely, sulfaclozine 30 mg (B) induced progressive histological damage in a dose- and time-dependent manner. Hence, hepatic tissue at a low dose showed dilatation and proliferation of the portal area and degenerative changes with hepatic vacuolation. Conversely sulfaclozine 70 mg (C, D), high doses led to the detection of hepatic necrosis and loss of hepatic organization, along with fibrosis and hypercellularity in the bile duct. At the same time, giving sulfaclozine along with Saccharomyces, whether in low (E) or high (F) doses, lessened the tissue damage, leading to some swelling in the portal area and a little bit of liver cell damage as shown in Figs. 10, 11 and 12, and 13.

#### Kidney

**Fig. 14:**
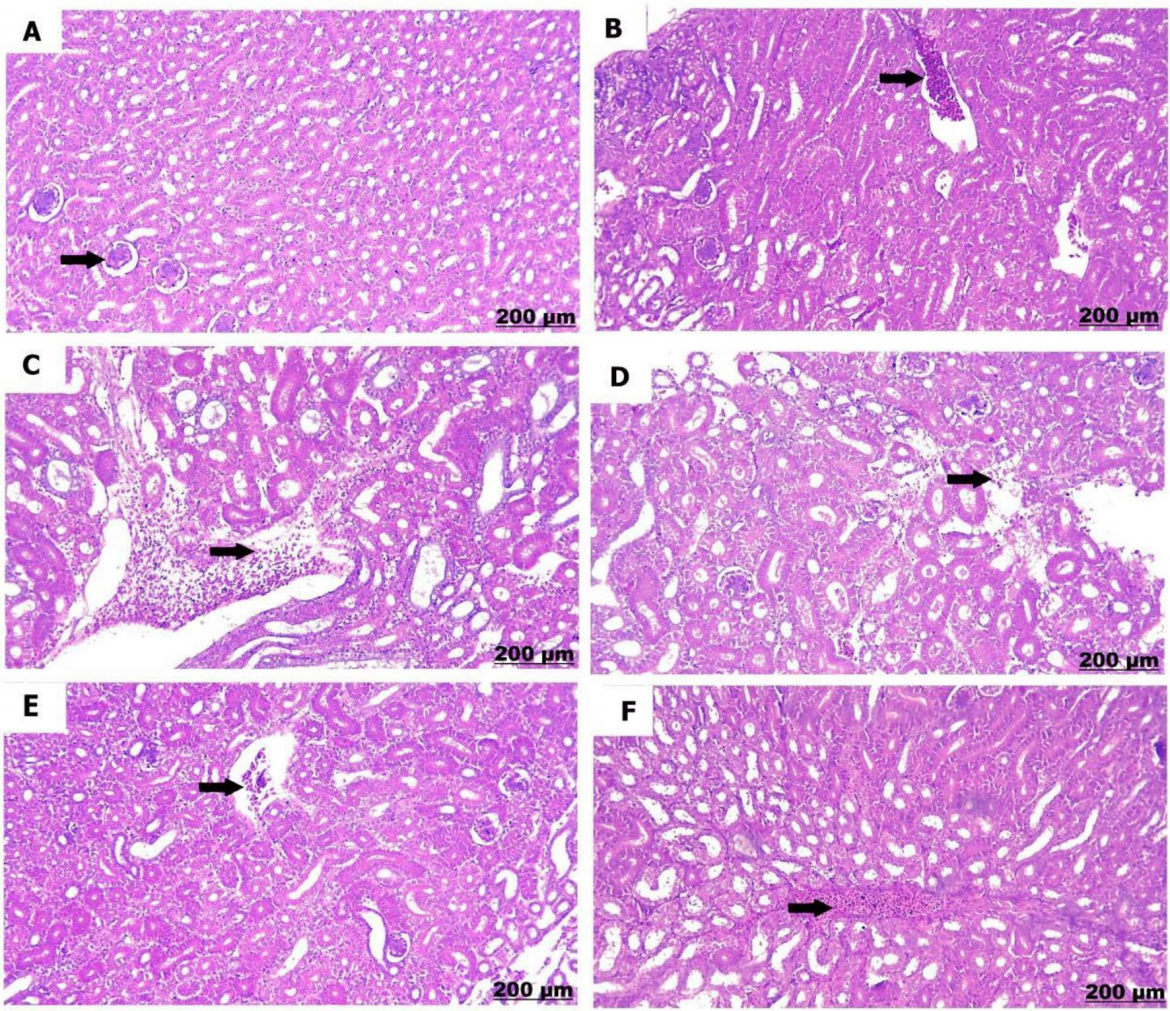
5 days post treatment. (**A**) shows intact glomeruli. (**B**) shows congestion of the renal vessels. (**C**) shows severe congestion and stagnation of blood vessels. (**D**) shows renal inflammation and edema. (**E**) shows minimally congested blood vessels. (**F**) shows moderate congestion of blood vessels

**Fig. 15:**
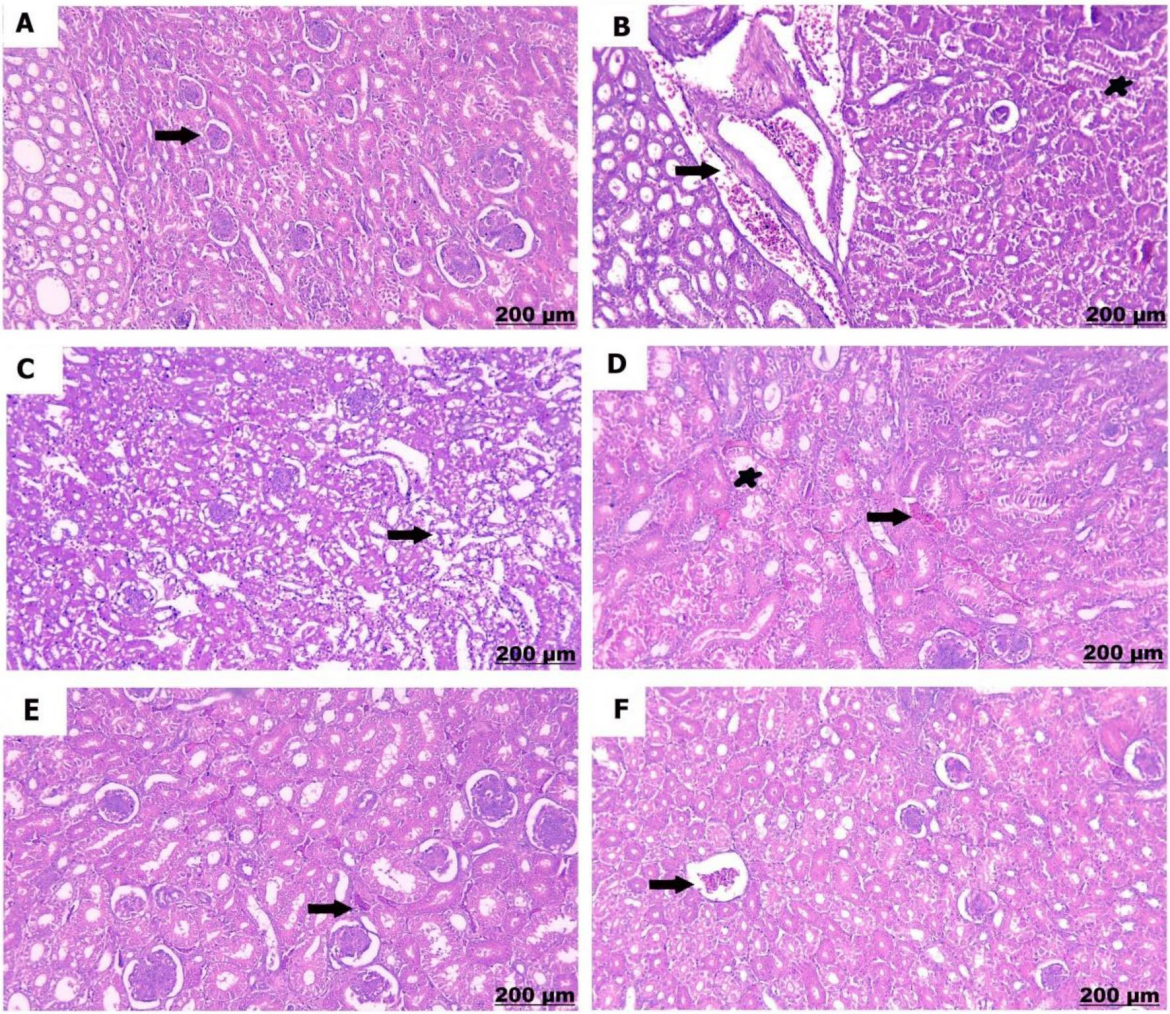
10 days post treatment. (**A**) shows normal arrangement of the glomeruli. (**B**) shows congestion and thickening of the renal vessels. (**C**) shows severe necrosis of the renal tubules. (**D**) shows renal congestion (arrow) and necrosis (star). (**E**) shows slight congested blood vessels. (**F**) shows moderate congestion of blood vessels

**Fig. 16:**
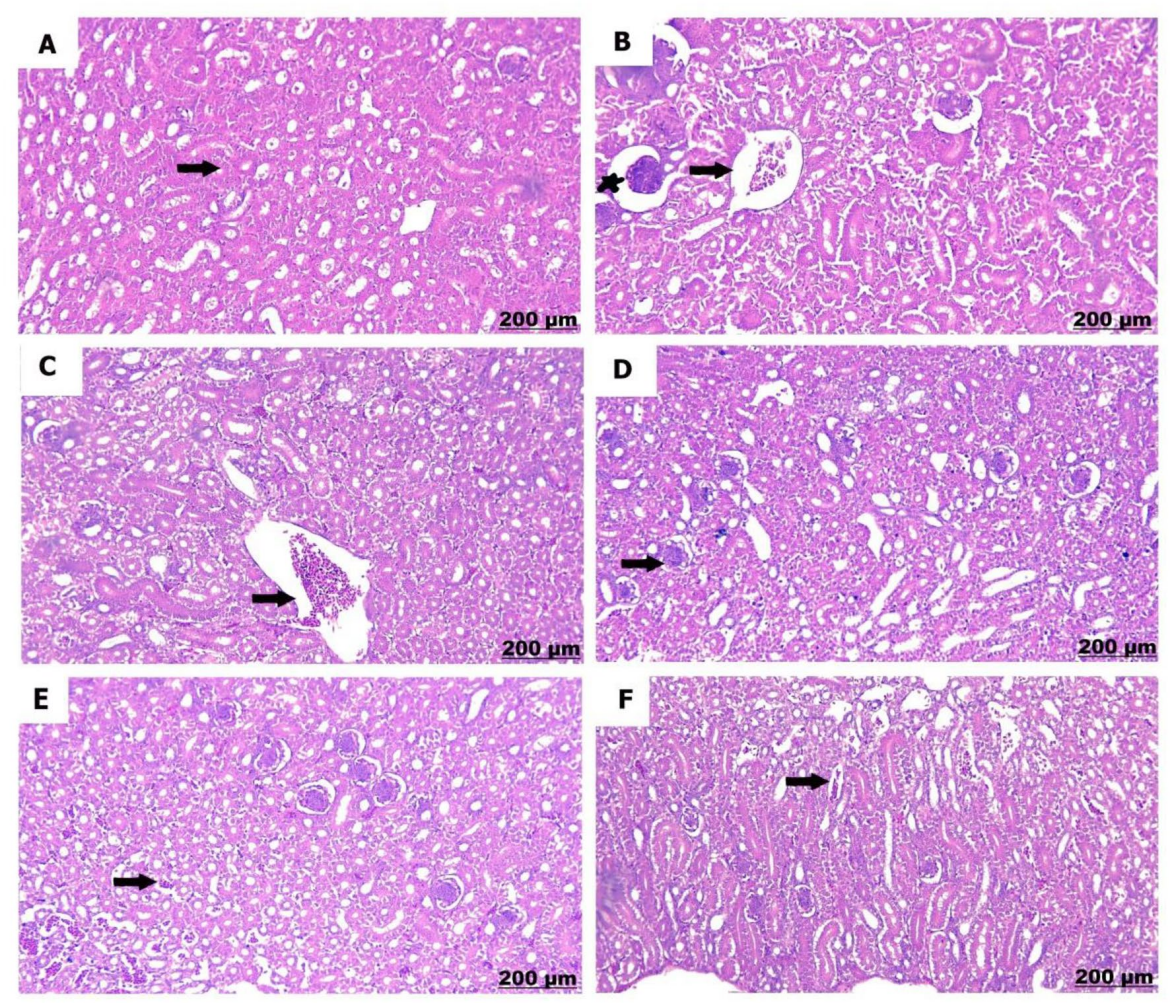
15 days post treatment. (**A**) shows normally arranged renal tubules. (**B**) shows congestion of the renal vessels (arrow), also expansion of bowman’s space (star). (**C**) shows prominent engorgement and dilatation of the renal vessels. (**D**) shows glomerular hypercellularity and distension. (**E**) shows mild congestion of the blood vessels. (**F**) shows modrate congestion and expansion of the blood vessels

**Fig. 17:**
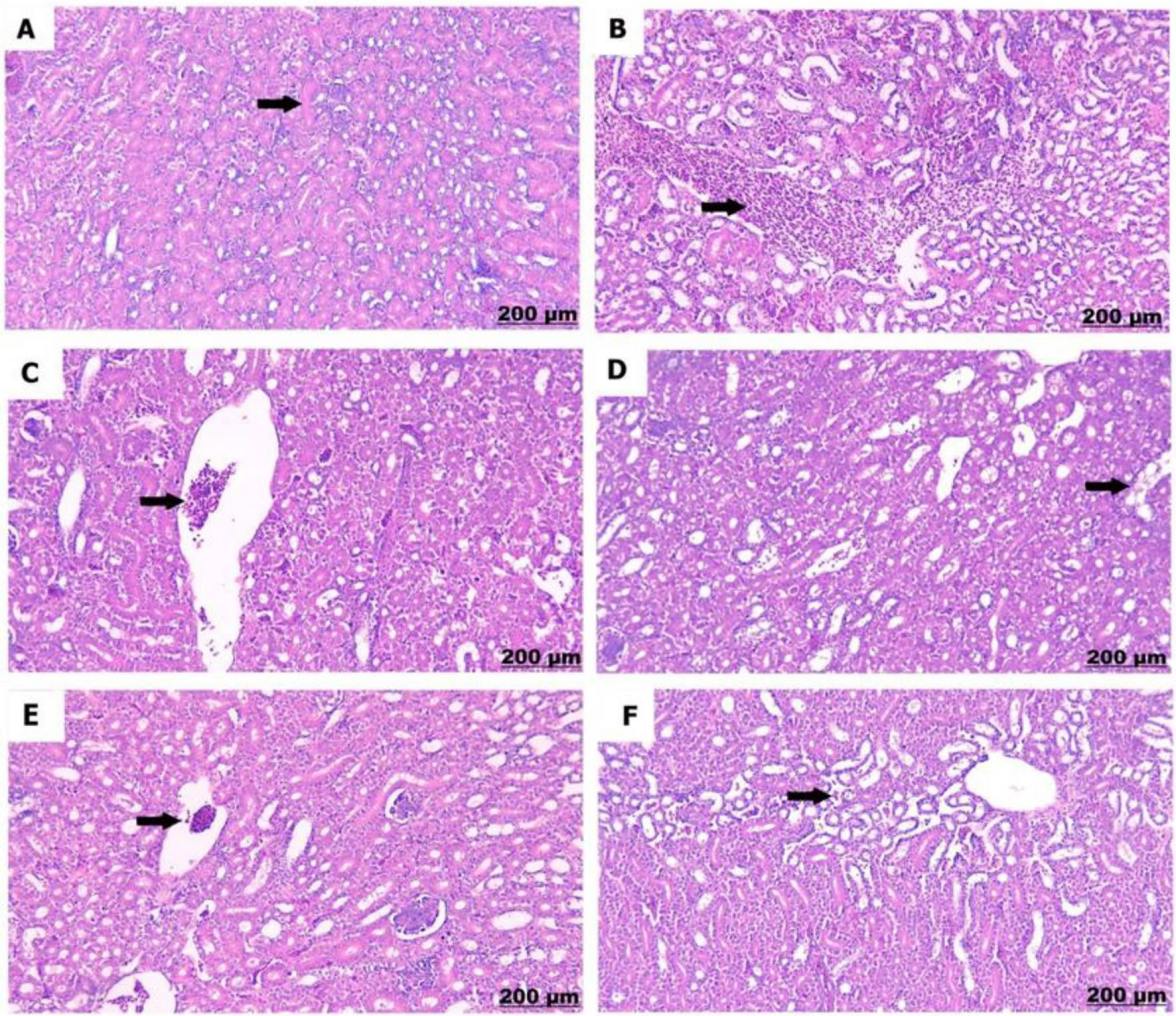
20 days post treatment. (**A**) shows intact renal tubules. (**B**) shows interstitial hemorrhage and congestion. **(C)** shows significant congestion of the renal vessels. **(D)** shows necrosis of the renal tubules. **(E)** shows mild congestion of the blood vessels. **(F)** shows mild interstitial hemorrhage and edema

The kidneys of the control chicks (A) showed normal structures with healthy glomeruli and properly organized renal tubules. However, a low dose of sulfaclozine (B) showed swollen and enlarged renal blood vessels, along with some inflammatory cells moving into the area. With high doses of sulfaclozine (C, D), the kidney tissue showed very swollen and congested blood vessels, serious damage to the kidney tubule cells, and large groups of lymphocytes. The altered histological architecture was attenuated after Saccharomyces administration (E & F); the renal tissue revealed mild congestion and dilatation of the renal blood vessels, mild vacuolation of hepatocytes, and infiltration of mononuclear cells in Figs. 14, 15, 16, and 17.

## Discussion

To determine the effects of administering intermediate (30 mg/kg) & high (70 mg/kg) doses of sulfacalozine to broiler chickens on body weight gain, various biochemical parameters, and the histopathologic changes in the kidney and liver, sulfonamide derivatives were widely used due to their low cost, ease of administration, and broad range of applications. Probiotics like yeast, which are non-digestible nutrients used as food supplements, encourage the growth of colon bacteria and offer the host positive, beneficial consequences [17].

In the current study, the control group’s body weight significantly declined compared to the sulfaclozine treatment groups (those receiving 30 or 70 mg) [18].This result supports our findings, which indicate that adding antibiotics to broiler diets did not increase the feed conversion ratio; instead, it decreased digestive efficiency by lowering absorptive surface areas and destroying digestive enzymes. On the other hand, antibiotics have been shown to save energy from tissue uptake, which can be used for growth or to improve nutrition absorption [19]. In contrast, sulfaclozine plus yeast treatment improved body weight, especially in Group 4. This aligns with the findings of Li et al., [20]. Probiotics have been shown to benefit the intestinal environment, food absorption, and growth performance in broiler chickens, significantly increasing the final body weight, as confirmed by Sobczak and Kozlowski [21]. These results also align with Neijat et al. [22], who observed higher body weights in ducklings treated with probiotics instead of an antibiotic-treated group. Some studies have found that probiotics can help break down parts of feed that the body can’t digest, which produces short-chain fatty acids that provide energy for the animal and improve its overall performance [23].

Furthermore, probiotics could boost the production of digestive enzymes in the gastrointestinal tract, facilitating the breakdown of complex feed ingredients for easier absorption [24]. Probiotics enhance the absorption of nutrients, such as vitamins and minerals, by promoting the expression of nutrient transporters in the intestinal lining, which improves growth performance and organ weight [25]. Furthermore, according to research, probiotics may change gut microbiota composition, boosting intestinal health and nutrient absorption, which may affect cholesterol [26].

### Biochemical parameters

We found that G3 was more negatively affected by sulfaclozine medications than the 2nd group, which results in an immunological allergic reaction that causes serious hepatocyte damage. Chemical substances such as carbon tetrachloride, antibiotics, and chemotherapeutics cause oxidative stress, leading to higher levels of AST and ALT than G1 [27]. Similar findings were reported by Verma and Kaplowitz [28], who showed that sulfanilamides are targeted for metabolism in the liver; when liver damage occurs; sulfa medicines raise the levels of ALT and AST enzymes in the extracellular fluid and plasma. The G4 and 5th groups significantly decreased ALT and AST levels. S. cerevisiae maintained the histological features of *L. ramada* liver by regulating ALT and AST activity. Similarly, Akanmu et al. [29] discovered that dietary S. cerevisiae reduced ALT and AST activities. S. cerevisia*e* plays a protective role by reducing oxidative stress. This aligns with the findings of Haque et al., [30], who found that the probiotic-supplemented groups had a lower AST value than the control group. When baker’s yeast was added to the diet of rabbits [31] and broilers [32] ALT and AST levels decreased. Similarly, as a prophylactic approach [33], discovered that probiotics dramatically lowered blood ALT and AST activity.

Probiotics help the body make more helpful enzymes, reduce harmful processes, and create vitamins or substances that fight germs, enhancing liver function and boosting immunity in broilers, leading to better productivity, as noted by Hassanein and Soliman [34]. By improving liver function, probiotics may also reduce the negative effects of an unbalanced gut flora [35]. Serum creatinine, a breakdown product of creatinine phosphate in the muscle, is usually produced by the body at a steady rate depending on muscle mass. With the potential to improve liver function, probiotics may lessen the adverse consequences caused by an imbalance in the gut flora [35]. The body typically creates serum creatinine, the breakdown product of creatinine phosphate in muscle, at a constant rate dependent on muscle mass. It is fully removed from the bloodstream via glomerular filtration and proximal tubular secretion. The gold standard for renal function, creatinine clearance, which controls the GFR, was calculated using serum creatinine levels [36]. Chickens and uric acid are the primary end products of protein metabolism that are excreted through the kidneys, feces, or sweat [37].Since it is well-known that changes in blood levels of creatinine and uric acid indicate kidney problems, our results showed that the groups treated with sulfaclozine (the second and third groups) had much higher kidney enzyme levels than the control group.

Sulfaclozine harms the kidneys by increasing the levels of creatinine and uric acid in the blood, which can lead to kidney damage, and this is why these levels go up [38]. Probiotic bacteria can use urea, creatinine, uric acid, and other toxic substances as growth nutrients [39]. Such an increase may explain the protective effect of yeast on the kidneys of the fourth and fifth groups in the current study. Proteins aid in the production of hormones, enzymes, and other compounds, as well as in the formation and repair of tissues. Proteins are essential components of blood, muscles, bones, and skin. A decrease in the amount of protein may inhibit its production. Serum protein levels show how well the liver can make proteins and drop when the liver is damaged [40], which happens after liver injury.

Our investigation revealed that sulfaclozine’s effects on total protein and albumin levels were significantly decreased in the second and third groups. This drop could be attributed to antibiotic stress or a reduction in protein synthesis, consistent with Al-Rikaby et al., [41]. On the other hand, there were notable increases in total protein and albumin in the yeast-treated groups, which is consistent with Adriani et al., [42], who discussed how probiotics raise blood protein and albumin levels, which affects the growth rate of broiler chickens and plays a major role in the deposition of protein into meat. Attia et al., [43], also confirmed this finding, stating that *S. cerevisiae* at 1.5% significantly increased total plasma protein, albumin, and globulin.

### Oxidative status

Too many reactive oxygen species (ROS) lead to oxidative stress, which can damage important molecules like proteins, fats, and DNA when the body can’t keep up with the removal and repair of this damage due to too much ROS produced by the mitochondria [44]. The group treated with sulfaclozine only showed higher levels of serum AST and ALT, which are signs of liver cell damage related to increase MDA, a substance produced when fats break down that indicates oxidative damage. Also, the levels of antioxidant genes like superoxide dismutase (SOD), catalase (CAT), and glutathione peroxidase (GPX) might be changed by exposure to outside substances like antibiotics [45]. These results agree with Zhou’s et al., research [46], which showed that higher doses of sulfamethoxazole reduce the activities of SOD, CAT, and GSH-Px, which are related to increased ROS production. Furthermore, Hu et al., [47] discovered that after 30 days of treatment with 100 µg/L sulfamethoxazole, the treated group had much lower levels of CAT, glutathione, and SOD activity compared to the control group, while MDA levels went up, showing later signs of oxidative stress and cell damage. Supplementation with *S. cerevisiae* increased CAT levels and decreased MDA levels in the fourth and fifth groups. This lends credence to the notion that yeast contributes to broiler antioxidant properties. These outcomes are consistent with the research of Gong et al., [48], who found that adding dietary yeast at 1.5% or 3.0% improved their antioxidant status. The capacity of *S. cerevisiae* to function as an antioxidant likely contributes to enhanced immunity in the intestines and throughout the body. Glycans, vitamins, proteins, and amino acids are just a few active components that provide *S. cerevisiae* with antioxidant properties [49].

Certain types of oxidative stress can lead to the production of TGFβ1 during cell death and inflammation, which starts the creation of ROS and harms the extracellular matrix (ECM). TGFβ1 can also reduce the amounts of antioxidants such as glutaredoxin, catalase, glutathione peroxidase (GPx), superoxide dismutase (SOD), and glutathione (GSH), which help manage ROS activity [50]. Our current study found a link between TGFβ1 production and oxidative stress in the group treated with sulfaclozine. These findings are consistent with those of Kurakzua et al., [51]. Probiotics can inhibit the activity of enzymes that produce free radicals. Matušková et al., [52] identified cytochrome P450 (CYP) enzymes, cyclooxygenase (COX) [53] and the NOX complex [54]. Moreover, studies have linked probiotics to metabolic problems and cellular demise [55].

Furthermore, probiotics regulate inflammation and immunological responses via histone acetylation [56]. Research shows that probiotics can lower inflammation and oxidative stress [57] and may help treat liver damage by reducing TGFβ1 levels [58]. When sulfaclozine and *S. cerevisiae* were used in the fourth and fifth experimental groups, TGFβ1 levels dropped significantly and decreased cell responses. Recent research has revealed that probiotics can reduce oxidative stress caused by experimental TGF-β1levels [59]. Numerous studies have suggested that TGF-β signaling can be blocked by the NRF2 transcription factor, which is necessary for the phase II response [60]. NRF2 may suppress TGF-β1 signaling by promoting antioxidant enzyme production [61]. This constituent with Won et al., [62] who stated that using probiotics in induced liver fibrosis of mouse model down- regulate TGF-β1. The second and third groups of chickens in our experiment were administered sulfaclozine alone, which disrupts NRF2 regulation. Antibiotics enhance the production of ROS and lipid peroxidation, as demonstrated by Yu et al., [63] and [64] who also discovered that synthetic drugs, especially those in the third group, impair NRF2 regulation. Additionally, it significantly reduced the expression of NRF2, which is associated with a decline in the activity of antioxidant enzymes, such as CAT, GPx, and SOD. Based on our investigations, broilers that received sulfaclozine in combination with Saccharomyces cerevisiae had considerably higher NRF2 levels than broilers administered sulfaclozine alone. This finding is consistent with Wang et al., [65] who found that probiotics increased NRf-2 mRNA expression, suggesting improved antioxidant function. The level of reactive oxygen species in the body can be decreased by probiotics because they can strengthen the body’s defenses against disease and prevent tissue damage [66].

### Histopathology

Numerous in vitro and in vivo studies have linked oxidative stress to the onset and aggravation of liver fibrosis. In almost all long-term liver diseases, excessive oxygen species break down important molecules, directly activate hepatic stellate cells, release substances that encourage fibrosis, and harm liver cells. These procedures ultimately cause liver damage and development of hepatic fibrosis [67]. Bile duct hypercellularity, hepatic necrosis, and loss of hepatic organization were evident in the results of the second and third groups, which were administered only sulfaclozine. These findings were consistent with those of Odigie [68], who reported histological changes in the liver and kidney of albino Wistar rats after they were administered prophylactic sulfa.

However, the liver segments of the groups treated with sulfaclozine and S. cerevisiae (4th and 5th days) demonstrated improvement in liver architecture, with mild hepatocyte disintegration and moderate portal region congestion. Tilg and Hotamisligil [33] reported that probiotics have hepatoprotective effects on liver function and similar results supported their findings. Histological sections of broiler renal tissues treated with sulfaclozine alone showed severe congestion, dilatation of blood vessels, and severe necrosis of the renal tubular epithelium. Similar findings align with Singh and Kumar [27] who showed that the administration of sulfanilamide caused renal impairment.

## Conclusion

The results indicated that adding *Saccharomyces cerevisiae* 1.5 g/L before administering sulfaclozine 30 mg rather than 70 mg reduced the drug’s harmful effects, protecting the liver and kidneys, which are crucial for birds’ growth and health.

### Limitation

More research is required to determine whether Saccharomyces cerevisiae can improve the growth performance and immunity of chicken species while reducing the harmful effects of various antibiotics and treating metabolic diseases.

## Electronic supplementary material

Below is the link to the electronic supplementary material.

Supplementary Material 1

Supplementary Material 2

Supplementary Material 3

Supplementary Material 4

Supplementary Material 5

Supplementary Material 6

Supplementary Material 7

Supplementary Material 8

## Data Availability

No datasets were generated or analysed during the current study.

## Abbreviations

MDA: Malondialdehyde
AST: Aspartate transaminase
ALT: Alanine transaminase
TGFβ1: Transforming growth factor b1
NRF2: Nuclear factor erythroid 2 related factor2
S.: cerevisiae Saccharomyces cerevisiae
ROS: Reactive oxygen species
SOD: Superoxide dismutase
GPX: Glutathione peroxidase
CYP450: Cytochrome P450
COX: Cyclooxygenase
NOX: NADPH oxidase
