# Supplementary figures and images for: Saccharomyces cervisiae ameliorative impact combined with sulfaclozine on broiler chicken oxidative status
Source: BMC Vet Res. 2025 Aug 6;21:507. doi: 10.1186/s12917-025-04955-x (PMC12326629; doi:10.1186/s12917-025-04955-x)

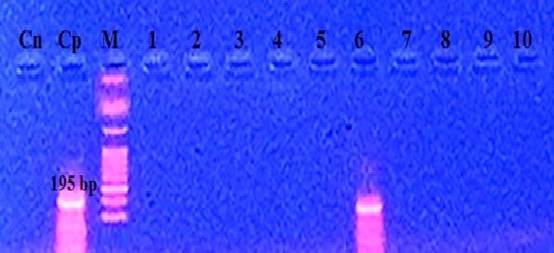

Supplement: Supplementary file 1 — Supplementary Material 1 [file 12917_2025_4955_MOESM1_ESM.png]

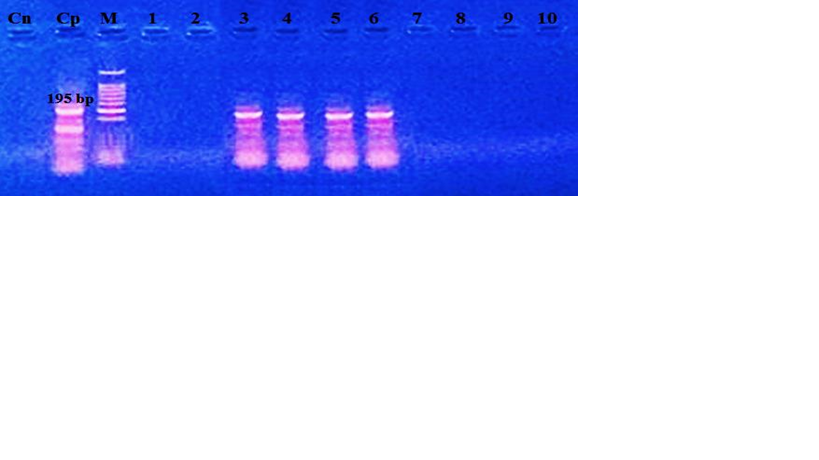

Supplement: Supplementary file 2 — Supplementary Material 2 [file 12917_2025_4955_MOESM2_ESM.png]

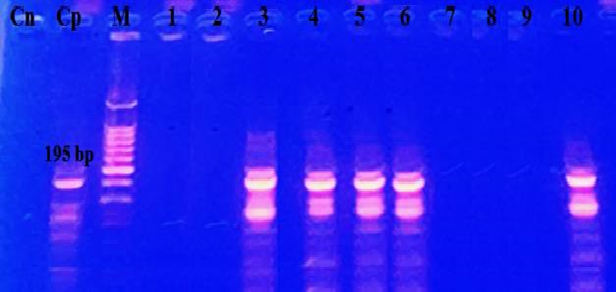

Supplement: Supplementary file 3 — Supplementary Material 3 [file 12917_2025_4955_MOESM3_ESM.png]

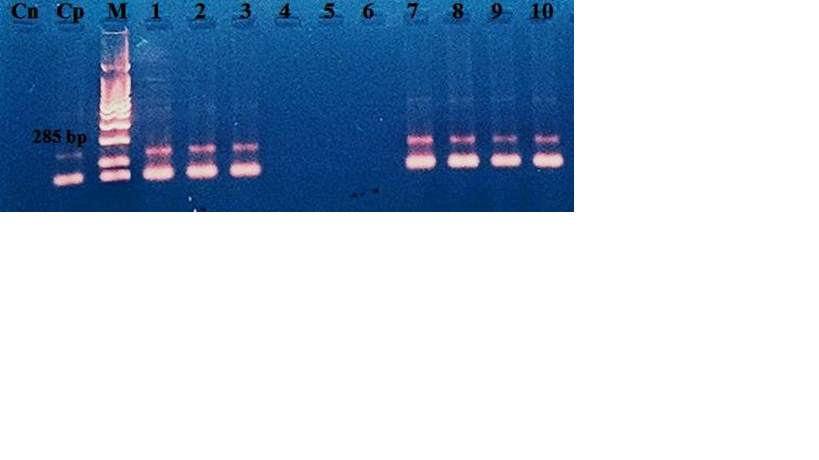

Supplement: Supplementary file 4 — Supplementary Material 4 [file 12917_2025_4955_MOESM4_ESM.png]

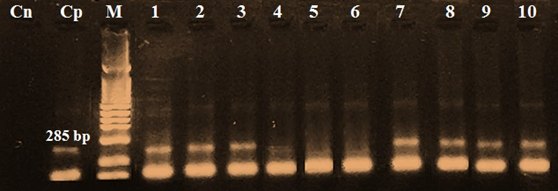

Supplement: Supplementary file 5 — Supplementary Material 5 [file 12917_2025_4955_MOESM5_ESM.png]

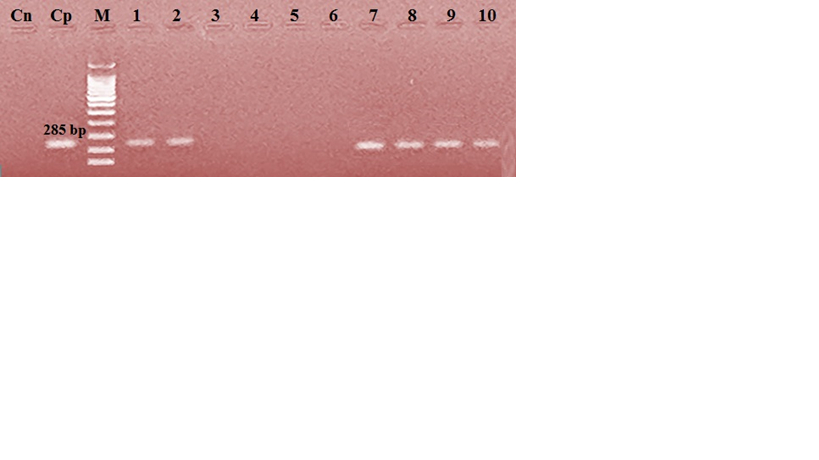

Supplement: Supplementary file 6 — Supplementary Material 6 [file 12917_2025_4955_MOESM6_ESM.png]

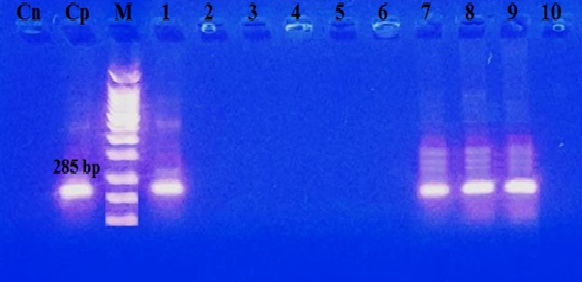

Supplement: Supplementary file 7 — Supplementary Material 7 [file 12917_2025_4955_MOESM7_ESM.png]

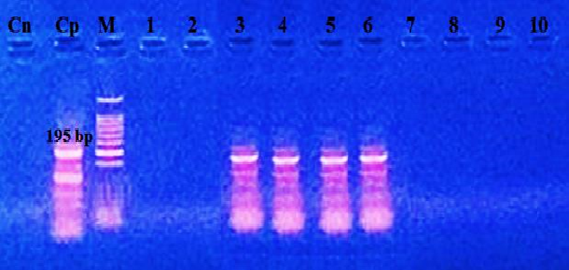

Supplement: Supplementary file 8 — Supplementary Material 8 [file 12917_2025_4955_MOESM8_ESM.png]
